# Reduced Intracellular c-di-GMP Content Increases Expression of Quorum Sensing-Regulated Genes in *Pseudomonas aeruginosa*

**DOI:** 10.3389/fcimb.2017.00451

**Published:** 2017-10-17

**Authors:** Song Lin Chua, Yang Liu, Yingying Li, Hui Jun Ting, Gurjeet S. Kohli, Zhao Cai, Pipob Suwanchaikasem, Kelvin Kau Kit Goh, Sean Pin Ng, Tim Tolker-Nielsen, Liang Yang, Michael Givskov

**Affiliations:** ^1^Lee Kong Chian School of Medicine, Nanyang Technological University, Singapore, Singapore; ^2^Singapore Centre for Environmental Life Sciences Engineering, Nanyang Technological University, Singapore, Singapore; ^3^School of Biological Sciences, Nanyang Technological University, Singapore, Singapore; ^4^Department of Immunology and Microbiology, Costerton Biofilm Center, University of Copenhagen, Copenhagen, Denmark

**Keywords:** *Pseudomonas aeruginosa*, cyclic-di-GMP, quorum sensing, PQS, rhamnolipids

## Abstract

Cyclic-di-GMP (c-di-GMP) is an intracellular secondary messenger which controls the biofilm life cycle in many bacterial species. High intracellular c-di-GMP content enhances biofilm formation via the reduction of motility and production of biofilm matrix, while low c-di-GMP content in biofilm cells leads to increased motility and biofilm dispersal. While the effect of high c-di-GMP levels on bacterial lifestyles is well studied, the physiology of cells at low c-di-GMP levels remains unclear. Here, we showed that *Pseudomonas aeruginosa* cells with high and low intracellular c-di-GMP contents possessed distinct transcriptome profiles. There were 535 genes being upregulated and 432 genes downregulated in cells with low c-di-GMP, as compared to cells with high c-di-GMP. Interestingly, both *rhl* and *pqs* quorum-sensing (QS) operons were expressed at higher levels in cells with low intracellular c-di-GMP content compared with cells with higher c-di-GMP content. The induced expression of *pqs* and *rhl* QS required a functional PqsR, the transcriptional regulator of *pqs* QS. Next, we observed increased production of *pqs* and *rhl*-regulated virulence factors, such as pyocyanin and rhamnolipids, in *P. aeruginosa* cells with low c-di-GMP levels, conferring them with increased intracellular survival rates and cytotoxicity against murine macrophages. Hence, our data suggested that low intracellular c-di-GMP levels in bacteria could induce QS-regulated virulence, in particular rhamnolipids that cripple the cellular components of the innate immune system.

## Introduction

*Pseudomonas aeruginosa* can cause opportunistic infections in humans, such as cystic fibrosis lung infections, burn wounds and urinary tract infections (Bodey et al., 1983). This is attributed to its ability to form biofilms and produce an abundance of virulence factors to impair the host immune response (Bjarnsholt et al., 2009; Fazli et al., 2011).

Similar to many Gram-negative bacteria species, the biofilm and planktonic lifestyles in *P. aeruginosa* are controlled by the secondary messenger bis-(3′-5′)-cyclic-dimeric-GMP (c-di-GMP) (Romling et al., 2005). C-di-GMP is synthesized by diguanylate cyclases (DGCs) and degraded by phosphodiesterases (PDEs) (Hengge, 2009). High intracellular c-di-GMP content enhances biofilm formation, whereas low intracellular c-di-GMP content leads to biofilm dispersal and the return to planktonic phase (Hisert et al., 2005; Romling et al., 2005; Kulasakara et al., 2006; Chua et al., 2014; Yu et al., 2015). The redundancy of DGC and PDE genes in the genome confers *P. aeruginosa* the survival advantage to respond to various stresses from the environment. For instance, the *wspR* DGC is important in the sensing of reactive oxygen species (ROS) and formation of biofilms resilient to ROS stress (Chua et al., 2016a).

Another system that plays important roles in biofilm formation and virulence is quorum sensing (QS), which is the intercellular communication system positively dependent on cell density and QS autoinducer (AI) concentrations (Fuqua et al., 1994; Whitehead et al., 2001; Ng and Bassler, 2009). *P. aeruginosa* possesses four major QS systems, encoded by the *las, rhl, pqs* and *iqs* systems, with the *las* and *rhl* systems employing homoserine lactones, namely the *N*-(3-oxododecanoyl)-homoserine lactone (OdDHL) and N-butanoyl-L-homoserine lactone (BHL) respectively as their AIs (Gambello and Iglewski, 1991; Passador et al., 1993; Ochsner and Reiser, 1995; Pearson et al., 1995), while *pqs* and *iqs* sytems using the 2-heptyl-3-hydroxy-4(1H)-quinolone (PQS) and 2-(2-hydroxyphenyl)-thiazole-4-carbaldehyde respectively (Cao et al., 2001; Diggle et al., 2007; Lee et al., 2013). The AIs will bind and activate the transcriptional regulators, resulting in the transcription of downstream QS operons. The QS systems interregulate one another, notably the *las* system and *pqs* system activate the *rhl* system (Pesci et al., 1997; McKnight et al., 2000; Farrow et al., 2008). The QS systems control the production of many virulence factors, such as pyocyanin (by the *pqs* operon) and rhamnolipids (by the *rhl* operon) (Pearson et al., 1997; Xiao et al., 2006). The rhamnolipids are biosurfactants which are highly cytotoxic to eukaryotic cells, as previously demonstrated by the induction of *rhl* operon- controlled gene expression in biofilm bacteria exposed to polymorphonuclear leukocytes (PMNs) and subsequent destruction of these important defensive immune cells (Alhede et al., 2009).

While the impact of high c-di-GMP content on biofilm formation is well understood, the consequences of low intracellular c-di-GMP content other than biofilm dispersal remain unclear. Our previous study showed that freshly dispersed cells, during the short-term liberation process, appeared to be highly virulent as compared to biofilm cells (Chua et al., 2014). It remains elusive whether reduced c-di-GMP content may have a long-term impact on bacterial physiology and virulence.

Hence, we aimed to investigate the impact of low vs. high c-di-GMP concentrations on *P. aeruginosa* virulence mechanisms. We compared the transcriptomes of *P. aeruginosa* PAO1 cells “locked” in a condition with high c-di-GMP content (by using the *wspF* mutation to induce constitutive expression of WspR) and the cells “locked” in a condition with low c-di-GMP content (by over expressing the YhJH PDE) cultivated under the similar growth conditions. As the WspF protein is the inhibitor of the WspR DGC, the *wspF* mutation will cause expression of WspR, thereby promoting the synthesis of c-di-GMP leading to high internal levels (Hickman et al., 2005). The PAO1/p_lac_-*yhjH* strain contains the constitutively expressed PDE gene *yhjH* leading to low internal levels of c-di-GMP, a condition important in swarming and swimming motility (Pesavento et al., 2008; Chua et al., 2013).

We found that low intracellular c-di-GMP content induced expression of the QS systems, specifically the *rhl* and *pqs* systems, which led to increased production of several virulence factors, such as rhamnolipids and pyocyanin. This was correlated to increased killing of macrophages. We showed that the induction of *rhl* and *pqs* QS under conditions of low c-di-GMP levels, was mediated by PqsR, the transcriptional regulator of *pqs* QS.

Hence, our present study suggested that c-di-GMP-governed biofilm dispersal might liberate bacteria capable of producing virulence factors, so as to survive and protect themselves from the phagocytic immune cells in the host. Hence, as a strategy to prevent the dissemination of biofilm infections, the use of QS inhibitors (Hentzer et al., 2003) can potentially reduce the production of QS-related virulence factors.

## Materials and methods

### Strains, plasmids, and growth conditions

The bacterial strains and plasmids used in this study are listed in Table S1. *Escherichia coli* DH5α strain was used for standard DNA manipulations (Bertani, 1951). LB medium was used to cultivate *E. coli* strains. Batch cultivation of *Pseudomonas aeruginosa* strains was carried out at 37°C in ABT minimal medium (Clark, 1968) with 5 g L^−1^ glucose (ABTG) or 2 g L^−1^ glucose and 2 g L^−1^ casamino acids (ABTGC). To maintain plasmids in *E. coli*, 2 ml LB was supplemented with 100 μg ampicillin (Ap) mL^−1^, 15 μg mL^−1^ gentamicin (Gm), 15 μg mL^−1^ tetracycline (Tc), or 8 μg mL^−1^ chloramphenicol (Cm). In *P. aeruginosa*, 30 μg mL^−1^ Gm, 50 μg mL^−1^ Tc, and 200 μg carbenicillin mL^−1^ (Cb) were used for marker selection.

### Quantification of c-di-GMP

Bacterial cells in 5 ml ABTGC were harvested and pelleted by centrifugation at 13,000 g for 3 min. The supernatant was removed and the cell pellet was immediately snap-frozen in liquid nitrogen. The cell pellet was re-suspended in 1 ml of acetonitrile/methanol/water (40:40:20) mixture. An aliquot of cells (10 μl) was used for protein quantification. The cells were then lysed with a probe tip ultrasonicator (Amplitude 30%; 5 s ON, 5 s OFF) for 1 min in ice slurry. The cell debris was removed by centrifugation at 13000 g for 3 min. The liquid phase was then evaporated by using the vacuum concentrator, leaving behind the nucleotide precipitate. The samples were then re-suspended in 100 μl ddH_2_O and centrifuged at 10,000 g, 4°C for 10 min. The solutions were transferred to glass vials and injected through liquid chromatography- mass spectrometry (LCMS).

For the LC portion, the samples in the glass vials were run through the BEH C18 (1.7 μm; 2.1 × 50 mm) column with injection volume of 5 μl at 0.3 ml min^−1^ for a total runtime of 6 min, with the mobile phase A as 10 mM ammonium formate in water + 0.1% formic acid and mobile phase B as methanol + 0.1% formic acid. For the MS portion, the samples were then analyzed by Xevo TQ-S, Waters mass spectrometer, under the ESI positive ion mode (capillary voltage: 3.8 kV, desolvation temperature: 400°C). The cyclic di-GMP compound was detected by monitoring ion transition of 691.2 m/z to 152.0 m/z at collision energy 36 eV.

For protein quantification, the cell aliquot was treated in 1 ml of 5 M NaOH at 95°C for 5 min. After cooling the samples for 15 mins, the proteins were processed with the Qubit® protein assay kit (NanoOrange dye) and quantified by the Qubit® 2.0 Fluorometer (Invitrogen). The concentration of c-di-GMP was then normalized with protein quantity. Experiments were performed in triplicate, and results were shown as the mean ± s.d.

### Quantification of PDE activity

Bacterial cells in 5 ml ABTGC were harvested and pelleted by centrifugation at 13,000 g for 3 min. The supernatant was removed and the cell pellet was resuspended in 5 ml 0.9% NaCl. The cells were lysed with a probe tip ultrasonicator (Amplitude 30%; 5 s ON, 5 s OFF) for 3 min in ice slurry to obtain a crude extract. As previously described (Kuchma et al., 2007), the crude extracts were incubated with 5 ml of 5 mM bis(p-nitrophenyl) phosphate (bis-pNPP) in buffer (5 mM MgCl_2_, 50 mM Tris-HCl [pH 9.3], 50 mM NaCl). The release of p-nitrophenol was quantified by using a microplate reader (Tecan Infinite Series 2000) at OD410 every 15 min for 16 h.

As described in the previous section, protein concentration was determined by the Qubit® 2.0 Fluorometer (Invitrogen).The PDE activity was then normalized with protein quantity. Experiments were performed in triplicate, and results were shown as the mean ± s.d.

### RNA preparation

PAO1, PAO1ΔwspF and PAO1/p_*lac*_*-yhjH* were grown in 1 ml ABTGC in each well (triplicates) within a 24-well microplate (Nunc) for 7 h till late logarithmic phase in 37°C, 200 rpm shaking. Bacterial cells were first treated with RNA Protect (Qiagen, Netherlands) and then treated with lysozyme. Total RNA was extracted using RNeasy Mini Kit (Qiagen, Netherlands). On-column DNase digestion with the RNase-free DNase Set (Qiagen) was used to remove DNA. The DNA contamination levels were assessed by using the Qubit®dsDNA High Sensitivity (HS) assay (PicoGreen dye) and the Qubit® 2.0 Fluorometer (Invitrogen). The integrity of total RNA was assessed by using the Bioanalyser RNA analysis kit (Agilent Technologies) and the Agilent 2100 Bioanalyzer (Agilent Technologies). The Ribo-Zero™ Magnetic Kit (Bacteria) (Epicentre) was used to deplete 16S, 23S, and 5S rRNAs from the samples.

### RNA sequencing and data analysis

Gene expression analysis of 2 biological replicates was conducted by RNA-Seq technology (Illumina). The RNA was fragmented to 200–300 bp fragments using divalent cations under elevated temperature.

First and second strand cDNA were then synthesized and treated by end repair and adapter ligation. After the 12-cycle PCR enrichment, the quality of the libraries was assessed using the Agilent 2100 Bioanalyzer (Agilent Technologies). The libraries were sequenced using the Illumina HiSeq2000 platform with paired-end protocol and read lengths of 100 nt.

The sequence reads were assembled and analyzed by “RNA-Seq and expression analysis” application of CLC genomics Workbench 6.0 (CLC Bio, Aarhus, Denmark). The PAO1 genome (http://www.ncbi.nlm.nih.gov/nuccore/110645304) was utilized as the reference genome. The following criteria were used to filter the unique sequence reads: maximum number of hits for a read of 1, minimum length fraction of 0.9, minimum similarity fraction of 0.8, and maximum number of two mismatches. Genes were annotated with Pseudomonas Genome Database (Winsor et al., 2011). The mapping results of RNA-Seq raw data from CLC genomics Workbench 6.0 were subjected to DESeq2 package for statistical analysis (Anders and Huber, 2010) by reading them into R/Bioconductor (Gentleman et al., 2004). The transcript counts were normalized to the effective library size. Hierarchical clustering analysis was performed and a heatmap was drawn for the 1000 most highly expressed genes of PAO1, PAO1ΔwspF, and PAO1/p_*lac*_*-yhjH* using heatmap.2 package of R/Bioconductor (Gentleman et al., 2004). Furthermore, the normalized counts were stabilized according their variance as outlined in the DESeq2 package tutorial and a principle component analysis (PCA) plot was generated. The differentially expressed genes among PAO1, PAO1Δ*wspF*, and PAO1/ p_*lac*_*-yhjH* were identified by performing a negative binomial test using the DESeq2 package of R/Bioconductor. Transcripts were stringently determined as differentially expressed when having a fold change larger than 5 and an adjusted *p*-value smaller than 0.05.

Accession number for the RNA-seq is PRJNA381683.

### qRT-PCR analysis

Total RNA from cells grown in 2 ml ABTGC was extracted using RNeasy Mini Kit (Qiagen) with on- column DNase digestion. The concentration and purity of the extracted RNA were measured by NanoDrop 2000 spectrophotometer (Thermo Scientific), while the integrity of RNA was analyzed by Agilent 2200 TapeStation System (Agilent Technologies). The elimination of contaminating DNA was confirmed via real time PCR amplification of the *rpoD* gene with total RNA as template.

First-strand cDNA was first synthesized from total RNA with the SuperScript® III First-Strand Synthesis SuperMix kit (Invitrogen). The cDNA was used as template for qRT-PCR with a kit of SYBR® Select Master Mix (Applied Biosystems, Life Technologies) on the StepOnePlus Real-Time PCR System (Applied Biosystems, Life Technologies). The gene *rpoD* was used as endogenous control. To verify specific single-product amplification, melting curves were analyzed.

### Quantification of BHL by using *ΔlasIΔrhlI/P_*rhlA*_-gfp* reporter fusion

Supernatants (2 ml) from *P. aeruginosa* strains grown in ABTGC in 37°C overnight were filtered through 0.2-μm filters, and the filtrates were collected. Overnight culture of the reporter strain Δ*lasI*Δ*rhlI/p*_*rhlA*_*-gfp* was adjusted to OD_600_ = 0.2 using ABTGC medium. 100 μl of filtrate was added to 100 μl of Δ*lasI*Δ*rhlI/p*_*rhlA*_*-gfp* in a 96-well plate (Nunc, Denmark). Because Δ*lasI*Δ*rhlI* does not produce BHL, *p*_*rhlA*_*-gfp* was induced by the addition of serial diluted filtrates containing BHL. GFP fluorescence from *p*_*rhlA*_*-gfp* expression (expressed in relative fluorescence units, RFU) was measured for each well using a microplate reader (Tecan Infinite 2000) and was normalized to the OD_600_ of each well. Experiments were performed in triplicate, and results are shown as the mean ± s.d.

### Quantification of PQS by using *ΔpqsA/p_*pqsA*_-gfp* reporter fusion

Supernatants (2 ml) from *P. aeruginosa* strains grown in ABTGC in 37°C overnight filtered through 0.2-μm filters and the filtrates were collected. Overnight culture of the reporter strain Δ*pqsA*/p_*pqsA*_*-gfp* was adjusted to OD_600_ = 0.2 using ABTGC medium. 100 μl of filtrate was added to 100 μl of Δ*pqsA*/ p_*pqsA*_*-gfp* in a 96-well plate (Nunc, Denmark). Because Δ*pqsA* does not produce PQS, p_*pqsA*_*-gfp* was induced by the addition of serial diluted filtrates containing PQS. GFP fluorescence from p_*pqsA*_*-gfp* expression (expressed in relative fluorescence units, RFU) was measured for each well using a microplate reader (Tecan Infinite 2000) and was normalized to the OD_600_ of each well. Experiments were performed in triplicate, and results are shown as the mean ± s.d.

### Quantification of rhamnolipids by orcinol assay

Relative amounts of rhamnolipids produced by *P. aeruginosa* strains were quantified as previously described (Wittgens et al., 2011; Fong et al., 2016). The supernatant of strain grown in 2 ml ABTGC in 37°C overnight was filtered with 0.2-μm filter and treated with equal volumes of ethyl acetate. Samples were then mixed with vortexing for 30 s, with a phase separation by putting samples briefly in centrifuge for 30 s at 5,000 g. The upper organic phase containing rhamnolipids were transferred to new tube. The organic solvent was then removed by evaporation with a vacuum concentrator. The residue containing rhamnolipids was then re-suspended in 100 μl ddH_2_O. 100 μl of sample was mixed with 100 μl of 1.6% orcinol in ddH_2_O and 800 μl sulphuric acid (60% v/v). The samples were then incubated at 80°C for 30 mins and mixed periodically. After cooling the samples to room temperature for 15 mins, OD_420_ was measured by the microplate reader (Tecan Infinite 2000). Experiments were performed in triplicates and results were shown as mean ± standard deviation.

### Quantification of pyocyanin

Relative levels of pyocyanin produced by *P. aeruginosa* strains were quantified as previously described (Frank and Demoss, 1959; Fong et al., 2016). The supernatant of strain grown in 2 ml ABTGC in 37°C overnight was filtered with 0.2-μm filter and treated with equal volumes of chloroform. Samples were then mixed with vortexing for 30 s, with a phase separation by putting samples in centrifuge for 30 s at 5,000 g. The lower organic phase containing pyocyanin was transferred to new tube. 200 μl of 0.1 M hydrochloric acid (HCl) was then added to the chloroform phase and mixed with vortexing for 30 s, with a phase separation by putting samples briefly in centrifuge. The pink coloration which subsequently formed by acidification of pyocyanin, in the HCl phase at the top layer was then transferred to a 96-well microplate (Nunc, Denmark) and OD_500_ was measured by the microplate reader (Tecan Infinite 2000). Experiments were performed in triplicates and results were shown as mean ± standard deviation.

### Macrophages

The murine macrophage cell line RAW264.7 (ATCC No. TIB-71) was grown in 15 ml Dulbecco's Modified Eagle's Medium (DMEM) (Life Technologies), supplemented with 10% fetal bovine serum (FBS) (Gibco). Cells were incubated in 75 cm^2^ cell culture flasks (Nunc, Denmark) at a density of 5.0 × 10^6^ cells ml^−1^ for 72 h, at 37°C, 5% CO_2_ and 90% humidity.

The cells were checked against *Mycoplasma* contamination by using the PCR *Mycoplasma* detection kit (Abmgood, USA) before experiments.

### Macrophage cytotoxicity assay

To test the ability of cells to kill macrophages, cytotoxicity of macrophages was determined by monitoring cell integrity in 1 ml DMEM + 10% FBS + 20 μM propidium iodide (PI), as previously described (Chua et al., 2016b). Cells that stained by PI under the epifluorecent microscopy (Zeiss) with 20 × objective were observed as dead. The ratio of dead cells to live cells, enumerated from five images (each image contained approximately 200 macrophages), was then calculated. Experiments were performed in triplicates and results were shown as mean ± standard deviation.

## Results

### Comparing the c-di-GMP content of PAO1 cells with high and low c-di-GMP levels

**Figure 1:**
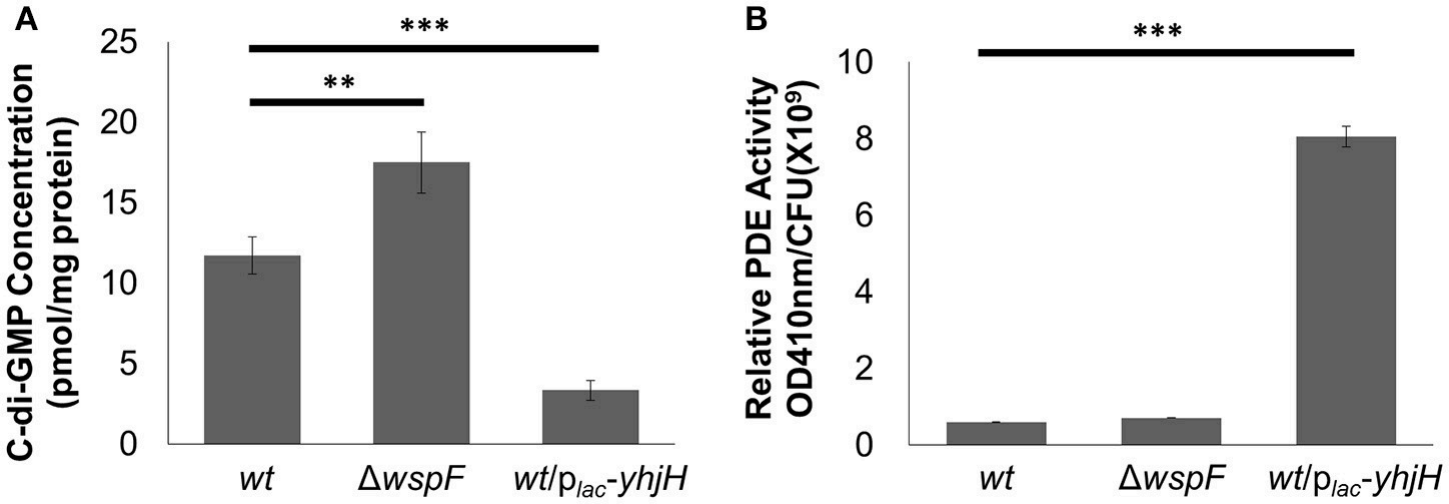
Higher PDE activity in *P. aeruginosa*/p_*lac*_*-yhjH* correlated to lower c-di-GMP levels. **(A)** C-di-GMP quantification by LCMS in *P. aeruginosa wt*, Δ*wspF* and *P. aeruginosa*/p_*lac*_*-yhjH*. **(B)** PDE activity in *P.aeruginosa wt*, Δ*wspF* and *P. aeruginosa*/p_*lac*_*-yhjH*. Means and s.d. from triplicate experiments are shown. ^**^P < 0.01, ^***^P < 0.001, Student's *t*-test.

We used RNA-sequencing to compare the transcriptomes of *P. aeruginosa* PAO1 cells “locked” in conditions of either high or low intracellular c-di-GMP contents. The late log phase *P. aeruginosa* Δ*wspF* mutant cells possessed a high intracellular c-di-GMP content due to the constitutively expressed WspR DGC protein (D'argenio et al., 2002; Rybtke et al., 2012; Chua et al., 2016a; Figure 1A), contributing to the overproduction of exopolysaccharides and low motility in Δ*wspF* cells. Hence, we used Δ*wspF* cells to mimic the biofilm stage. On the other hand, the late log phase *P. aeruginosa* p_*lac*_*-yhjH* mutant cells contained reduced c-di-GMP content due to the constitutively expressed YhjH PDE protein (Gjermansen et al., 2010; Chua et al., 2013; Figure 1A), thus mimicking cells freshly dispersed from the biofilms. We also corroborated our findings by detecting increased enzymatic PDE activity in the p_*lac*_*-yhjH* harboring cells, as compared to wild type and Δ*wspF* mutant cells (Figure 1B).

### Comparing the transcriptomes of PAO1 cells with high and low c-di-GMP levels

**Figure 2:**
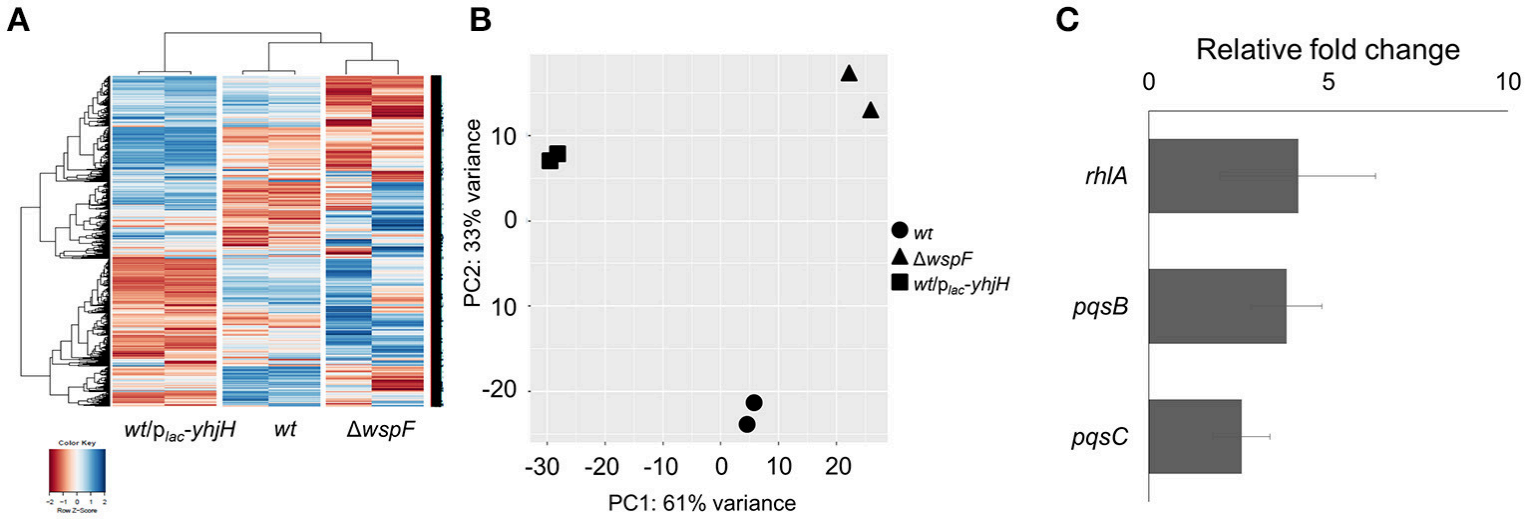
Comparison of gene expression by the *P. aeruginosa* wild-type, high intracellular c-di-GMP containing Δ*wspF* mutant and low intracellular c-di-GMP containing *P. aeruginosa*/p_*lac*_*-yhjH* mutant. **(A)** Heat map and **(B)** PCA plot comparison of wild-type, Δ*wspF* and *P. aeruginosa*/p_*lac*_*-yhjH*. **(C)** qRT-PCR analysis of *rhlA, pqsB* and *pqsC* expression in *P. aeruginosa*/p_*lac*_*-yhjH* culture relative to Δ*wspF* strain. Means and s.d. from triplicate experiments are shown.

**Figure 3:**
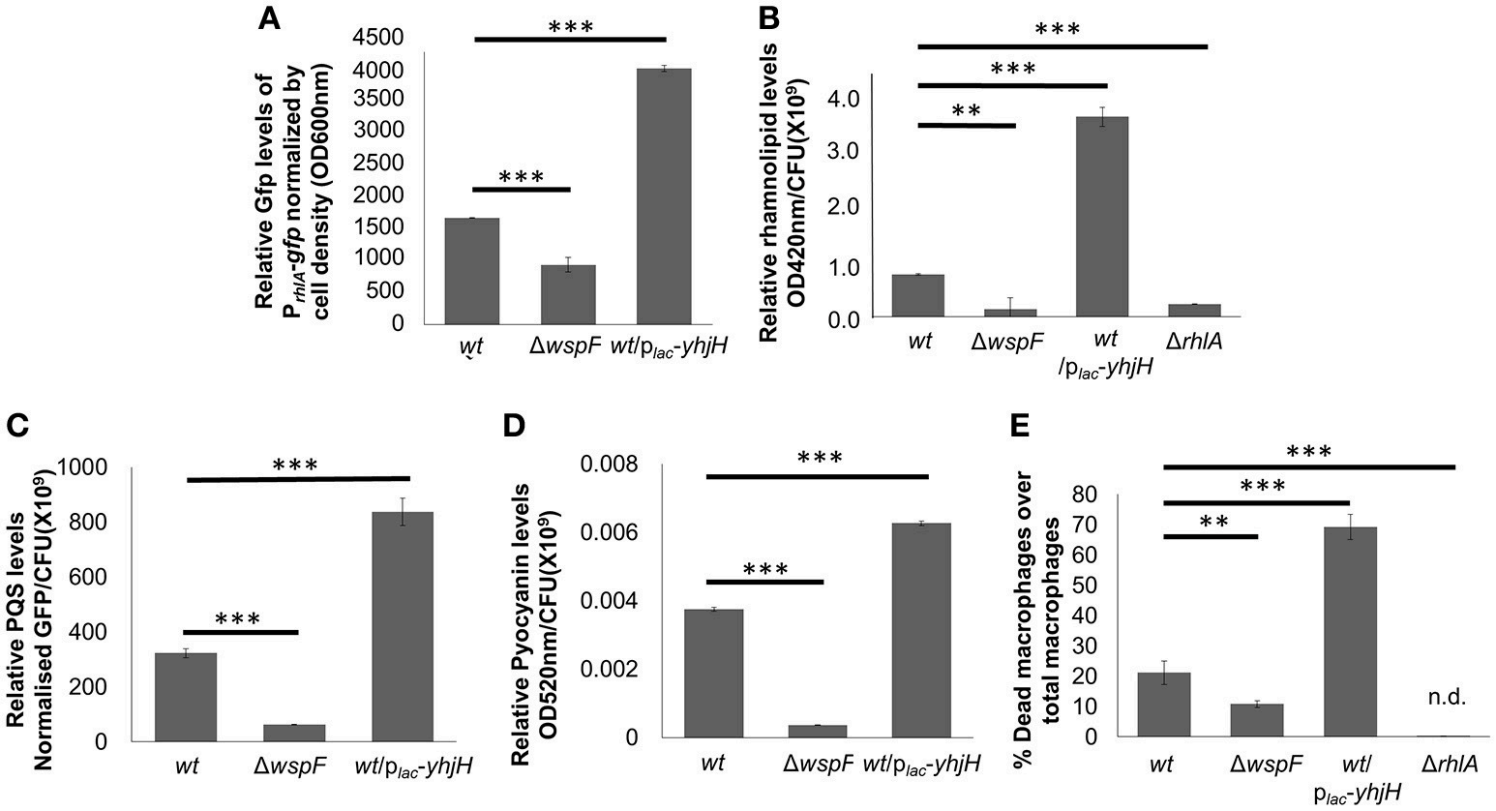
Induction of QS-controlled virulence factors in low intracellular c-di-GMP containing *P. aeruginosa*/p_*lac*_*-yhjH* mutant. Relative quantification of P_*rhlA*_-*ASV* **(A)**, rhamnolipids **(B)**, PQS **(C)** and pyocyanin **(D)** in PAO1, Δ*wspF* mutant and *P. aeruginosa*/p_*lac*_*-yhjH* mutant. **(E)** Cytotoxicity of *P. aeruginosa*/p_*lac*_*-yhjH* and Δ*wspF* to macrophages. Means and s.d. from triplicate experiments are shown. ^**^P < 0.01, ^***^P < 0.001, One-way ANOVA.

The Δ*wspF* and PAO1/p_*lac*_*-yhjH* mutants demonstrated distinct gene expression profiles according to the heat map diagram and PCA analysis (Figures 2A,B). 431 genes were up-regulated and 595 genes were down-regulated in the p_*lac*_*-yhjH* mutant as compared to the Δ*wspF* mutant (Data Sheet 1), including genes regulated by QS (in particular regulated by the *rhl* and *pqs* encoded systems). Specifically, *rhlR, rhlA* and *rhlB* were highly induced in the p_*lac*_*-yhjH* strain compared to the Δ*wspF* mutant (Data Sheet 1). This finding was validated by qRT-PCR analysis (Figure 2C) and the *rhl* QS reporter fusion p_*rhlA*_*-gfp* (Yang et al., 2009; Figure 3A). Accordingly, the p_*lac*_*-yhjH* strain was found to produce more rhamnolipids (Figure 3B), PQS (Figure 3C) and pyocyanin (Figure 3D) than the Δ*wspF* mutant, and was thus more cytotoxic to murine macrophages than the Δ*wspF* mutant (Figure 3E). This showed that the reduction of c-di-GMP levels lead to induction of the QS systems, which in turn stimulate production of a subset of QS-controlled virulence factors. Conversely, the Δ*wspF* mutant with the “mimicked” biofilm phenotype had higher expression of biofilm-related genes important for exopolysaccharide production (*pelB, pelC, pelD* and *PelF*) and siderophore production (*pvdD, pvdJ, pvdL, pvdO*), as compared to the PAO1/p_*lac*_*-yhjH* mutant.

### PqsR induces the pqs and rhl QS

**Figure 4:**
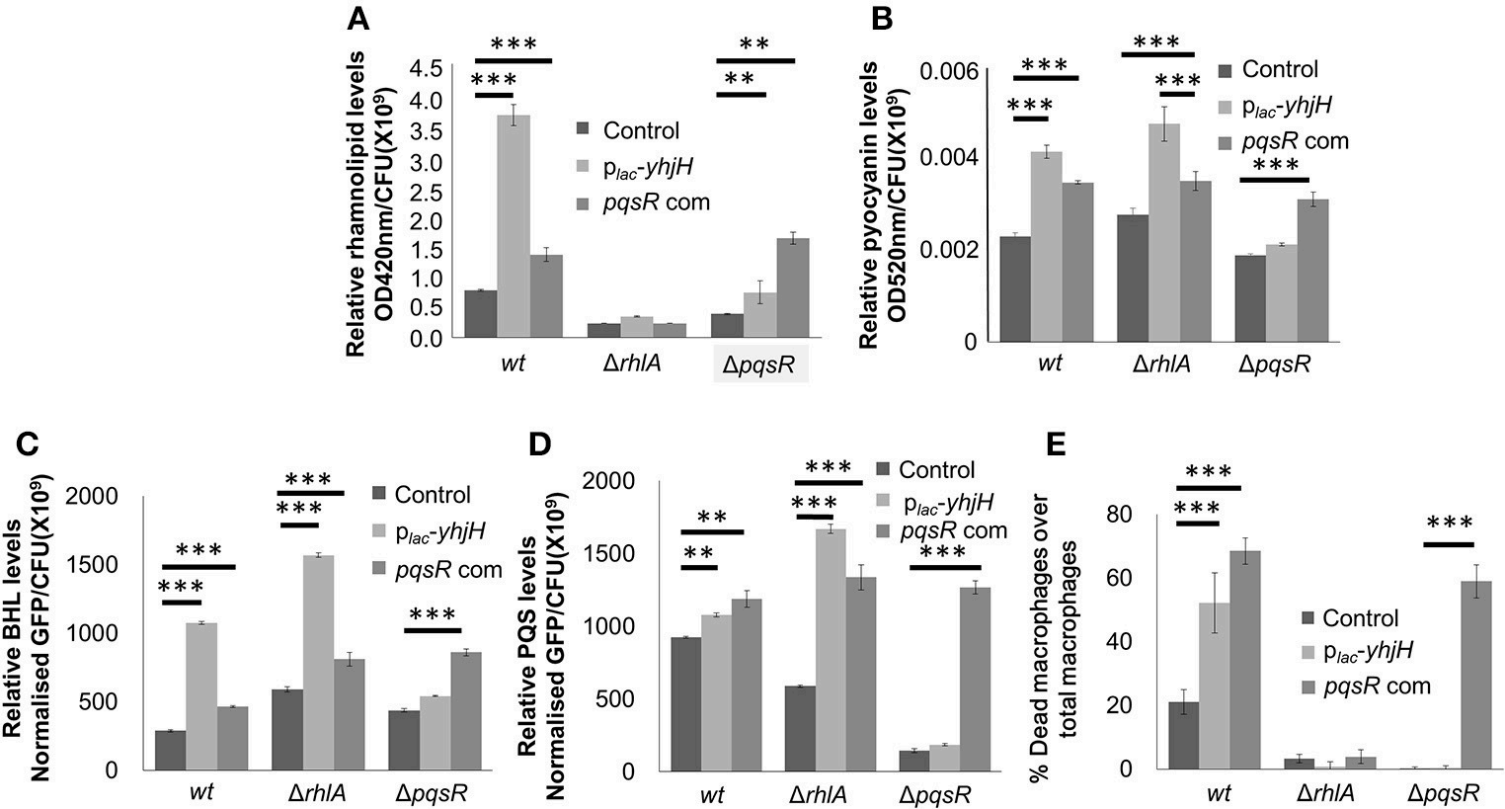
PqsR links c-di-GMP signaling to *rhl* quorum sensing. Rhamnolipid **(A)**, pyocyanin **(B)**, BHL **(C)** and PQS **(D)** quantification of *P. aeruginosa wt* and Δ*pqsR* cultures. **(E)** Cytotoxicity of *P.aeruginosa*/p_*lac*_*-yhjH* and Δ*pqsR* against macrophages. Means and s.d. from triplicate experiments are shown. ^**^P < 0.01, ^***^P < 0.001, One-way ANOVA.

We also noticed that genes from the *pqs* operon, such as *pqsA, pqsB, pqsC*, and *pqsH* were highly induced in cells with low intracellular c-di-GMP content (Data Sheet 1). The qRT-PCR analysis confirmed these findings (Figure 2C). Since the *pqs* operon is controlled by PqsR, we next hypothesized that PqsR could be crucial to the induction of *pqs* QS system under conditions of low c-di-GMP levels. Since the *rhl* encoded QS system can be induced by the *pqs* QS system (McKnight et al., 2000), it was also possible that activation of the *pqs* QS system boosted rhamnolipid production. Furthermore, a Δ*pqsR*/p_*lac*_*-yhjH* mutant produced lesser rhamnolipids than the p_*lac*_*-yhjH* mutant (Figure 4A). Accordingly, we showed that a PqsR deficient Δ*pqsR*/p_*lac*_*-yhjH* mutant expressed only low levels of pyocyanin, BHL and PQS compared with the p_*lac*_*-yhjH* mutant (Figures 4B–D). This correlated well with lower cytotoxicity to macrophages by Δ*pqsR* (Figure 4E). Hence, PqsR appeared to be a key regulator for induction of QS systems by low intracellular c-di-GMP content.

## Discussion

While most studies focused on the effects of high c-di-GMP levels on biofilm formation, there is a paucity of research on the physiology of cells undergoing conditions of low c-di-GMP signaling. Our previous study had shown that cells freshly dispersed from biofilms contained lower c-di-GMP levels than planktonic cells and biofilm cells (Chua et al., 2013), implying that dispersed cells possess a different physiology from biofilm and planktonic cells. This raised the question of how differing c-di-GMP levels impact the physiology in *P. aeruginosa*. As biofilm cells and dispersed cells had high physical and physiological heterogeneity (Stewart and Franklin, 2008), we used the Δ*wspF* and PAO1/p_*lac*_*-yhjH* mutants to imitate biofilm and dispersed cells respectively, and cultivated them as planktonic cultures which were easy to manipulate in controlled conditions.

In this work, we compared the transcriptomics of cells with high and low c-di-GMP levels. Other than biofilm dispersal, we had shown using transcriptomics that low c-di-GMP levels could lead to the induction of the *pqs* and *rhl* QS, with PqsR acting as mediator to activate both QS systems. Although we do not show that conditions of low c-di-GMP mediate increased PqsR, a previous study had shown that RsmA from the c-di-GMP-mediated Gac/Rsm pathway, was important in *pqs* and *rhl* QS (Burrowes et al., 2006). Hence, the result of activating both QS systems was the increased production of pyocyanin and rhamnolipids, which were correlated to higher virulence to immune cells. Interesting, it was previously observed that rhamnolipids acted as surfactant to facilitate biofilm cells to disperse from biofilms (Bhattacharjee et al., 2016).

Several research groups, such as ours (Chua et al., 2015; Yu et al., 2015) are currently investigating the possibility of exploiting the lowering of the c-di-GMP content in bacteria and dispersal as a biofilm control strategy. Our study had several implications for clinical and environmental applications of this biofilm dispersal strategy. Firstly, liberated bacterial cells could attain a unique physiological state if the c-di-GMP content is maintained at a lower level than planktonic cells and biofilm cells. This state could be reached after long-term growth of dispersed biofilm cells in the presence of agents that cause biofilm dispersal, thus warranting further studies on the biofilm-dispersed cells. Secondly, it appeared that a constitutively low c-di-GMP content renders the bacterial cells highly virulent, which might be essential for dispersed cells to survive the encounter with immune cells and cause development of sepsis.

Hence, it is important to evaluate the potential virulence outcome which applying c-di-GMP mediated biofilm dispersal during the eradication of biofilms, especially in infections. The use of QS inhibitors (Hentzer et al., 2003) can effectively negate the induction of QS pathways and production of virulence factors, to be used concurrently with c-di-GMP-mediated biofilm dispersal.

## Author contributions

SL and LY designed methods and experiments, analyzed the data, interpreted the results and wrote the paper. YL designed RNA-seq experiments, discussed analyses, interpretation, and presentation. YYL and HJ performed experiments for qRT-PCR work for analysis and interpretation. GK performed data processing for RNA-seq. SL, TT, LY and MG defined the research theme and discussed project outline. All authors have contributed to, seen and approved the manuscript.

### Conflict of interest statement

The authors declare that the research was conducted in the absence of any commercial or financial relationships that could be construed as a potential conflict of interest.

## References

[B1] Alhede M. Bjarnsholt T. Jensen P. O. Phipps R. K. Moser C. Christophersen L.. (2009). *Pseudomonas aeruginosa* recognizes and responds aggressively to the presence of polymorphonuclear leukocytes. Microbiology 155, 3500–3508. 10.1099/mic.0.031443-0 19643762

[B2] Anders S. Huber W. (2010). Differential expression analysis for sequence count data. Genome Biol. 11:R106. 10.1186/gb-2010-11-10-r106 20979621 PMC3218662

[B3] Bertani G. (1951). Studies on lysogenesis. I. The mode of phage liberation by lysogenic *Escherichia coli*. J. Bacteriol. 62, 293–300. 14888646 10.1128/jb.62.3.293-300.1951 PMC386127

[B4] Bhattacharjee A. Nusca T. D. Hochbaum A. I. (2016). Rhamnolipids mediate an interspecies biofilm dispersal signaling pathway. ACS Chem. Biol. 11, 3068–3076. 10.1021/acschembio.6b00750 27623227

[B5] Bjarnsholt T. Jensen P. O. Fiandaca M. J. Pedersen J. Hansen C. R. Andersen C. B.. (2009). *Pseudomonas aeruginosa* biofilms in the respiratory tract of cystic fibrosis patients. Pediatr. Pulmonol. 44, 547–558. 10.1002/ppul.21011 19418571

[B6] Bodey G. P. Bolivar R. Fainstein V. Jadeja L. (1983). Infections caused by *Pseudomonas aeruginosa*. Rev. Infect. Dis. 5, 279–313. 10.1093/clinids/5.2.279 6405475

[B7] Burrowes E. Baysse C. Adams C. O'gara F. (2006). Influence of the regulatory protein RsmA on cellular functions in *Pseudomonas aeruginosa* PAO1, as revealed by transcriptome analysis. Microbiology 152, 405–418. 10.1099/mic.0.28324-0 16436429

[B8] Cao H. Krishnan G. Goumnerov B. Tsongalis J. Tompkins R. Rahme L. G. (2001). A quorum sensing-associated virulence gene of *Pseudomonas aeruginosa* encodes a LysR-like transcription regulator with a unique self-regulatory mechanism. Proc. Natl. Acad. Sci. U.S.A. 98, 14613–14618. 10.1073/pnas.251465298 11724939 PMC64730

[B9] Chua S. L. Ding Y. Liu Y. Cai Z. Zhou J. Swarup S.. (2016a). Reactive oxygen species drive evolution of pro-biofilm variants in pathogens by modulating cyclic-di-GMP levels. Open Biol. 6:160162. 10.1098/rsob.160162 27881736 PMC5133437

[B10] Chua S. L. Hultqvist L. D. Yuan M. Rybtke M. Nielsen T. E. Givskov M.. (2015). *In vitro* and *in vivo* generation and characterization of *Pseudomonas aeruginosa* biofilm-dispersed cells via c-di-GMP manipulation. Nat. Protoc. 10, 1165–1180. 10.1038/nprot.2015.067 26158442

[B11] Chua S. L. Liu Y. Yam J. K. Chen Y. Vejborg R. M. Tan B. G.. (2014). Dispersed cells represent a distinct stage in the transition from bacterial biofilm to planktonic lifestyles. Nat. Commun. 5:4462. 10.1038/ncomms5462 25042103

[B12] Chua S. L. Tan S. Y. Rybtke M. T. Chen Y. Rice S. A. Kjelleberg S.. (2013). Bis-(3'-5')-cyclic dimeric GMP regulates antimicrobial peptide resistance in *Pseudomonas aeruginosa*. Antimicrob. Agents Chemother. 57, 2066–2075. 10.1128/AAC.02499-12 23403434 PMC3632963

[B13] Chua S. L. Yam J. K. Hao P. Adav S. S. Salido M. M. Liu Y.. (2016b). Selective labelling and eradication of antibiotic-tolerant bacterial populations in *Pseudomonas aeruginosa* biofilms. Nat. Commun. 7:10750. 10.1038/ncomms10750 26892159 PMC4762895

[B14] Clark D. J. (1968). Regulation of deoxyribonucleic acid replication and cell division in *Escherichia coli* B-r. J. Bacteriol. 96, 1214–1224. 4879557 10.1128/jb.96.4.1214-1224.1968 PMC252437

[B15] D'argenio D. A. Calfee M. W. Rainey P. B. Pesci E. C. (2002). Autolysis and autoaggregation in *Pseudomonas aeruginosa* colony morphology mutants. J. Bacteriol. 184, 6481–6489. 10.1128/JB.184.23.6481-6489.2002 12426335 PMC135425

[B16] Diggle S. P. Matthijs S. Wright V. J. Fletcher M. P. Chhabra S. R. Lamont I. L.. (2007). The *Pseudomonas aeruginosa* 4-quinolone signal molecules HHQ and PQS play multifunctional roles in quorum sensing and iron entrapment. Chem. Biol. 14, 87–96. 10.1016/j.chembiol.2006.11.014 17254955

[B17] Farrow J. M. III. Sund Z. M. Ellison M. L. Wade D. S. Coleman J. P. Pesci E. C. (2008). PqsE functions independently of PqsR-*Pseudomonas* quinolone signal and enhances the rhl quorum-sensing system. J. Bacteriol. 190, 7043–7051. 10.1128/JB.00753-08 18776012 PMC2580708

[B18] Fazli M. Bjarnsholt T. Kirketerp-Moller K. Jorgensen A. Andersen C. B. Givskov M.. (2011). Quantitative analysis of the cellular inflammatory response against biofilm bacteria in chronic wounds. Wound Repair Regen. 19, 387–391. 10.1111/j.1524-475X.2011.00681.x 21518086

[B19] Fong J. Yuan M. Jakobsen T. H. Mortensen K. T. Delos Santos M. M. Chua S. L.. (2016). Disulfide bond-containing ajoene analogues as novel quorum sensing inhibitors of *Pseudomonas aeruginosa*. J. Med. Chem. 60, 215–227. 10.1021/acs.jmedchem.6b01025 27977197

[B20] Frank L. H. Demoss R. D. (1959). On the biosynthesis of pyocyanine. J. Bacteriol. 77, 776–782. 13664660 10.1128/jb.77.6.776-782.1959 PMC290463

[B21] Fuqua W. C. Winans S. C. Greenberg E. P. (1994). Quorum sensing in bacteria: the LuxR-LuxI family of cell density-responsive transcriptional regulators. J. Bacteriol. 176, 269–275. 10.1128/jb.176.2.269-275.1994 8288518 PMC205046

[B22] Gambello M. J. Iglewski B. H. (1991). Cloning and characterization of the *Pseudomonas aeruginosa* lasR gene, a transcriptional activator of elastase expression. J. Bacteriol. 173, 3000–3009. 10.1128/jb.173.9.3000-3009.1991 1902216 PMC207884

[B23] Gentleman R. C. Carey V. J. Bates D. M. Bolstad B. Dettling M. Dudoit S.. (2004). Bioconductor: open software development for computational biology and bioinformatics. Genome Biol. 5:R80. 10.1186/gb-2004-5-10-r80 15461798 PMC545600

[B24] Gjermansen M. Nilsson M. Yang L. Tolker-Nielsen T. (2010). Characterization of starvation-induced dispersion in *Pseudomonas putida* biofilms: genetic elements and molecular mechanisms. Mol. Microbiol. 75, 815–826. 10.1111/j.1365-2958.2009.06793.x 19602146

[B25] Hengge R. (2009). Principles of c-di-GMP signalling in bacteria. Nat. Rev. Microbiol. 7, 263–273. 10.1038/nrmicro2109 19287449

[B26] Hentzer M. Wu H. Andersen J. B. Riedel K. Rasmussen T. B. Bagge N.. (2003). Attenuation of *Pseudomonas aeruginosa* virulence by quorum sensing inhibitors. EMBO J. 22, 3803–3815. 10.1093/emboj/cdg366 12881415 PMC169039

[B27] Hickman J. W. Tifrea D. F. Harwood C. S. (2005). A chemosensory system that regulates biofilm formation through modulation of cyclic diguanylate levels. Proc. Natl. Acad. Sci. U.S.A. 102, 14422–14427. 10.1073/pnas.0507170102 16186483 PMC1234902

[B28] Hisert K. B. Maccoss M. Shiloh M. U. Darwin K. H. Singh S. Jones R. A.. (2005). A glutamate-alanine-leucine (EAL) domain protein of *Salmonella* controls bacterial survival in mice, antioxidant defence and killing of macrophages: role of cyclic diGMP. Mol. Microbiol. 56, 1234–1245. 10.1111/j.1365-2958.2005.04632.x 15882417

[B29] Kuchma S. L. Brothers K. M. Merritt J. H. Liberati N. T. Ausubel F. M. O'toole G. A. (2007). BifA, a cyclic-Di-GMP phosphodiesterase, inversely regulates biofilm formation and swarming motility by *Pseudomonas aeruginosa* PA14. J. Bacteriol. 189, 8165–8178. 10.1128/JB.00586-07 17586641 PMC2168662

[B30] Kulasakara H. Lee V. Brencic A. Liberati N. Urbach J. Miyata S.. (2006). Analysis of *Pseudomonas aeruginosa* diguanylate cyclases and phosphodiesterases reveals a role for bis-(3'-5')-cyclic-GMP in virulence. Proc. Natl. Acad. Sci. U.S.A. 103, 2839–2844. 10.1073/pnas.0511090103 16477007 PMC1413825

[B31] Lee J. Wu J. Deng Y. Wang J. Wang C. Wang J.. (2013). A cell-cell communication signal integrates quorum sensing and stress response. Nat. Chem. Biol. 9, 339–343. 10.1038/nchembio.1225 23542643

[B32] McKnight S. L. Iglewski B. H. Pesci E. C. (2000). The Pseudomonas quinolone signal regulates rhl quorum sensing in *Pseudomonas aeruginosa*. J. Bacteriol. 182, 2702–2708. 10.1128/JB.182.10.2702-2708.2000 10781536 PMC101972

[B33] Ng W. L. Bassler B. L. (2009). Bacterial quorum-sensing network architectures. Annu. Rev. Genet. 43, 197–222. 10.1146/annurev-genet-102108-134304 19686078 PMC4313539

[B34] Ochsner U. A. Reiser J. (1995). Autoinducer-mediated regulation of rhamnolipid biosurfactant synthesis in *Pseudomonas aeruginosa*. Proc. Natl. Acad. Sci. U.S.A. 92, 6424–6428. 10.1073/pnas.92.14.6424 7604006 PMC41530

[B35] Passador L. Cook J. M. Gambello M. J. Rust L. Iglewski B. H. (1993). Expression of *Pseudomonas aeruginosa* virulence genes requires cell-to-cell communication. Science 260, 1127–1130. 10.1126/science.8493556 8493556

[B36] Pearson J. P. Passador L. Iglewski B. H. Greenberg E. P. (1995). A second N-acylhomoserine lactone signal produced by *Pseudomonas aeruginosa*. Proc. Natl. Acad. Sci. U.S.A. 92, 1490–1494. 10.1073/pnas.92.5.1490 7878006 PMC42545

[B37] Pearson J. P. Pesci E. C. Iglewski B. H. (1997). Roles of *Pseudomonas aeruginosa* las and rhl quorum-sensing systems in control of elastase and rhamnolipid biosynthesis genes. J. Bacteriol. 179, 5756–5767. 10.1128/jb.179.18.5756-5767.1997 9294432 PMC179464

[B38] Pesavento C. Becker G. Sommerfeldt N. Possling A. Tschowri N. Mehlis A.. (2008). Inverse regulatory coordination of motility and curli-mediated adhesion in *Escherichia coli*. Genes Dev. 22, 2434–2446. 10.1101/gad.475808 18765794 PMC2532929

[B39] Pesci E. C. Pearson J. P. Seed P. C. Iglewski B. H. (1997). Regulation of las and rhl quorum sensing in *Pseudomonas aeruginosa*. J. Bacteriol. 179, 3127–3132. 10.1128/jb.179.10.3127-3132.1997 9150205 PMC179088

[B40] Romling U. Gomelsky M. Galperin M. Y. (2005). C-di-GMP: the dawning of a novel bacterial signalling system. Mol. Microbiol. 57, 629–639. 10.1111/j.1365-2958.2005.04697.x 16045609

[B41] Rybtke M. T. Borlee B. R. Murakami K. Irie Y. Hentzer M. Nielsen T. E.. (2012). Fluorescence-based reporter for gauging cyclic di-GMP levels in *Pseudomonas aeruginosa*. Appl. Environ. Microbiol. 78, 5060–5069. 10.1128/AEM.00414-12 22582064 PMC3416407

[B42] Stewart P. S. Franklin M. J. (2008). Physiological heterogeneity in biofilms. Nat. Rev. Microbiol. 6, 199–210. 10.1038/nrmicro1838 18264116

[B43] Whitehead N. A. Barnard A. M. Slater H. Simpson N. J. Salmond G. P. (2001). Quorum-sensing in Gram-negative bacteria. FEMS Microbiol. Rev. 25, 365–404. 10.1111/j.1574-6976.2001.tb00583.x 11524130

[B44] Winsor G. L. Lam D. K. Fleming L. Lo R. Whiteside M. D. Yu N. Y.. (2011). *Pseudomonas genome* database: improved comparative analysis and population genomics capability for *Pseudomonas genomes*. Nucleic Acids Res. 39, D596–D600. 10.1093/nar/gkq869 20929876 PMC3013766

[B45] Wittgens A. Tiso T. Arndt T. T. Wenk P. Hemmerich J. Muller C.. (2011). Growth independent rhamnolipid production from glucose using the non-pathogenic *Pseudomonas putida* KT2440. Microb. Cell Fact. 10:80. 10.1186/1475-2859-10-80 21999513 PMC3258213

[B46] Xiao G. Deziel E. He J. Lepine F. Lesic B. Castonguay M. H.. (2006). MvfR, a key *Pseudomonas aeruginosa* pathogenicity LTTR-class regulatory protein, has dual ligands. Mol. Microbiol. 62, 1689–1699. 10.1111/j.1365-2958.2006.05462.x 17083468

[B47] Yang L. Rybtke M. T. Jakobsen T. H. Hentzer M. Bjarnsholt T. Givskov M.. (2009). Computer-aided identification of recognized drugs as *Pseudomonas aeruginosa* quorum-sensing inhibitors. Antimicrob. Agents Chemother. 53, 2432–2443. 10.1128/AAC.01283-08 19364871 PMC2687250

[B48] Yu S. Su T. Wu H. Liu S. Wang D. Zhao T.. (2015). PslG, a self-produced glycosyl hydrolase, triggers biofilm disassembly by disrupting exopolysaccharide matrix. Cell Res. 25, 1352–1367. 10.1038/cr.2015.129 26611635 PMC4670989

