# Supplementary material for: Reduced Intracellular c-di-GMP Content Increases Expression of Quorum Sensing-Regulated Genes in Pseudomonas aeruginosa
Source: Front Cell Infect Microbiol. 2017 Oct 17;7:451. doi: 10.3389/fcimb.2017.00451 (PMC5650985; doi:10.3389/fcimb.2017.00451)
Supplement: Supplementary file 1 [file Table1.PDF]

Table S1. Bacterial strains and plasmids used in the study.

Strain or plasmid	Relevant characteristics	Source or reference
P. aeruginosa		
PAO1	Prototypic non-mucoid wild-type strain	(1)
PAO1/Δ*wspF*	*wspF* knockout of PAO1 constructed by allelic exchange	(2)
PAO1/p$_{lac}$-*yhjH*	Tc^r; PAO1 containing the p$_{lac}$-*yhjH* vector	(3)
PAO1/p$_{rhlA}$-*gfp*	Gm^r; PAO1 containing the p$_{rhlA}$-*gfp* vector	(4)
PAO1/p$_{pqsA}$-*gfp*	Gm^r; PAO1 containing the p$_{pqsA}$-*gfp* vector	(5)
PAO1/Δ*rhlA*	*rhlA* knockout of PAO1 constructed by allelic exchange	(6)
PAO1/Δ*pqsR*	*pqsR* knockout of PAO1 constructed by allelic exchange	(7)
PAO1/Δ*lasI*/Δ*rhlI* /p$_{rhlA}$-*gfp*	Gm^r; PAO1/Δ*lasI*/Δ*rhlI* containing the p$_{rhlA}$-*gfp* vector	(8)
PAO1/Δ*pqsA*/p$_{pqsA}$-*gfp*	Gm^r; PAO1/Δ*pqsA* containing the p$_{pqsA}$-*gfp* vector	(9)
PAO1/Δ*pqsA*/p$_{lac}$-*yhjH*	Gm^r; PAO1/Δ*pqsA* containing the p$_{lac}$-*yhjH* vector	This study
E. coli		

DH5α	F–, ø80dlacZΔM15, Δ(lacZYA-argF)U169, deoR, recA1, endA1, hsdR17(rK–, mK+), phoA, supE44, λ–, thi-1,gyrA96, relA1	Laboratory Collection
Plasmids		
pUCP22	Ap^r Gm^r; broad-host-range cloning vector	(10)
pJN105	Gm^r; broad-host-range vector carrying the *araBAD* promoter	(11)
pMH305	Gm^r; pUCP22NotI-based expression vector carrying a promoterless *gfpmut3b** gene	(2)
pRK600	Cm^r; *ori* ColE1 RK2-Mob^+ RK2-Tra^+; helper vector for conjugation	(12)
p_{lac}-*yhjH*	Tc^r; pBBR1MCS3 carrying the *yhjH* gene	(13)
P_{BAD}-*yhjH*	Tc^r; pJN105 carrying the *yhjH* gene	(14)
p_{rhlA}-*gfp*	Ap^r Gm^r; pUCP22 carrying the p_{rhlA}-*gfp* fusion	(4)
P_{pqsA}-*gfp*	Ap^r Gm^r; pUCP22 carrying the p_{pqsA}-*gfp* fusion	(5)

References:

1. Holloway BW, Morgan AF. 1986. Genome organization in *Pseudomonas*. Annu Rev Microbiol 40:79-105.
2. Rybtke MT, Borlee BR, Murakami K, Irie Y, Hentzer M, Nielsen TE, Givskov M, Parsek MR, Tolker-Nielsen T. 2012. Fluorescence-based reporter for gauging cyclic di-GMP levels in *Pseudomonas aeruginosa*. Appl Environ Microbiol 78:5060-9.
3. Chua SL, Tan SY, Rybtke MT, Chen Y, Rice SA, Kjelleberg S, Tolker-Nielsen T, Yang L, Givskov M. 2013. Bis-(3'-5')-cyclic dimeric GMP regulates antimicrobial peptide resistance in *Pseudomonas aeruginosa*. Antimicrob Agents Chemother 57:2066-75.
4. Yang L, Nilsson M, Gjermansen M, Givskov M, Tolker-Nielsen T. 2009. Pyoverdine and PQS mediated subpopulation interactions involved in *Pseudomonas aeruginosa* biofilm formation. Mol Microbiol 74:1380-92.

5. Yang L, Barken KB, Skindersoe ME, Christensen AB, Givskov M, Tolker-Nielsen T. 2007. Effects of iron on DNA release and biofilm development by *Pseudomonas aeruginosa*. Microbiology 153:1318-28.
6. Alhede M, Bjarnsholt T, Jensen PO, Phipps RK, Moser C, Christophersen L, Christensen LD, van Gennip M, Parsek M, Hoiby N, Rasmussen TB, Givskov M. 2009. *Pseudomonas aeruginosa* recognizes and responds aggressively to the presence of polymorphonuclear leukocytes. Microbiology 155:3500-8.
7. D'Argenio DA, Calfee MW, Rainey PB, Pesci EC. 2002. Autolysis and autoaggregation in *Pseudomonas aeruginosa* colony morphology mutants. J Bacteriol 184:6481-9.
8. Yang L, Rybtke MT, Jakobsen TH, Hentzer M, Bjarnsholt T, Givskov M, Tolker-Nielsen T. 2009. Computer-aided identification of recognized drugs as *Pseudomonas aeruginosa* quorum-sensing inhibitors. Antimicrob Agents Chemother 53:2432-43.
9. Fletcher MP, Diggle SP, Camara M, Williams P. 2007. Biosensor-based assays for PQS, HHQ and related 2-alkyl-4-quinolone quorum sensing signal molecules. Nat Protoc 2:1254-62.
10. West SE, Schweizer HP, Dall C, Sample AK, Runyen-Janecky LJ. 1994. Construction of improved *Escherichia-Pseudomonas* shuttle vectors derived from pUC18/19 and sequence of the region required for their replication in Pseudomonas aeruginosa. Gene 148:81-6.
11. Newman JR, Fuqua C. 1999. Broad-host-range expression vectors that carry the L-arabinose-inducible Escherichia coli araBAD promoter and the araC regulator. Gene 227:197-203.
12. Kessler B, de Lorenzo V, Timmis KN. 1992. A general system to integrate lacZ fusions into the chromosomes of gram-negative eubacteria: regulation of the Pm promoter of the TOL plasmid studied with all controlling elements in monocopy. Mol Gen Genet 233:293-301.
13. Gjermansen M, Nilsson M, Yang L, Tolker-Nielsen T. 2010. Characterization of starvation-induced dispersion in *Pseudomonas putida* biofilms: genetic elements and molecular mechanisms. Mol Microbiol 75:815-26.
14. Chua SL, Hultqvist LD, Yuan M, Rybtke M, Nielsen TE, Givskov M, Tolker-Nielsen T, Yang L. 2015. In vitro and in vivo generation and characterization of *Pseudomonas aeruginosa* biofilm-dispersed cells via c-di-GMP manipulation. Nat Protoc 10:1165-80.
